# Preheating Influence on the Precipitation Microstructure, Mechanical and Corrosive Properties of Additively Built Al–Cu–Li Alloy Contrasted with Conventional (T83) Alloy

**DOI:** 10.3390/ma16144916

**Published:** 2023-07-10

**Authors:** Frank Adjei-Kyeremeh, Yudha Pratesa, Xiao Shen, Wenwen Song, Iris Raffeis, Uwe Vroomen, Daniela Zander, Andreas Bührig-Polaczek

**Affiliations:** 1 Foundry Institute, RWTH Aachen University, Intzestraße 5, 52072 Aachen, Germany; 2 Chair of Corrosion and Corrosion Protection, Foundry Institute, RWTH Aachen University, Intzestraße 5, 52072 Aachen, Germanyd.zander@gi.rwth-aachen.de (D.Z.); 3 Steel Institute, RWTH Aachen University, Intzestraße 1, 52072 Aachen, Germany; 4 Institute of Materials Engineering (IfW), University of Kassel, Moencheberg Str. 3, 34125 Kassel, Germany

**Keywords:** preheating, LPBF, T83, Al–Cu–Li, microstructure, SEM, APT, EBSD, precipitation, T_1_, corrosion, lower oxide resistance

## Abstract

In this paper, the high strength and lightweight Al–Cu–Li alloy (AA2099) is considered in as-built and preheated conditions (440 °C, 460 °C, 480 °C, 500 °C, and 520 °C). The purpose of this study is to investigate the influence of laser powder bed fusion (LPBF) in situ preheating on precipitation microstructure, mechanical and corrosive properties of LPBF-printed AA2099 alloy compared to the conventionally processed and heat-treated (T83) alloy. It is shown that precipitations evolve with increasing preheating temperatures from predominantly globular Cu-rich phases at lower temperatures (as-built, 440 °C) to more plate and rod-like precipitates (460 °C, 480 °C, 500 °C and 520 °C). Attendant increase with increasing preheating temperatures are the amount of low melting Cu-rich phases and precipitation-free zones (PFZ). Hardness of preheated LPBF samples peaks at 480 °C (93.6 HV0.1), and declines afterwards, although inferior to the T83 alloy (168.6 HV0.1). Preheated sample (500 °C) shows superior elongation (14.1%) compared to the T83 (11.3%) but falls short in tensile and yield strength properties. Potentiodynamic polarization results also show that increasing preheating temperature increases the corrosion current density (Icorr) and corrosion rate. Indicated by the lower oxide resistance (R_ox_), the Cu-rich phases compromise the integrity of the oxide layer.

## 1. Introduction

Research into AlLi alloys date back to the early 1900s when, what was later known as the ‘Friedlyander effect’ was discovered, where each 1 wt.%Li (up to 4 wt.%) when alloyed with aluminum was found to increase elastic modulus by 6 wt.% and decrease density by 3% [1]. Reduction in density of a Li-bearing alloy is promoted by an atomic weight of 6.94 compared to an Al of 26.98 [2,3]. Li addition increases the E-modulus, whether it is in solid solution or present as a second phase particle. In a solid solution, the magnitude of the elastic constants depends on interatomic interactions and potentials, while in a second phase, it depends on the intrinsic modulus of the second phase particle as well as its volume fraction. The lightweight benefits of Al–Li alloys have led to the development and deployment of 1st–3rd generation Al–Li alloys particularly for aerospace application [4].

Conventionally, these alloys are typically subjected to heat treatments, such as T6 temper conditions (solution treatment, and quenching plus artificial aging) or T8 conditions (solution treatment, cold working and artificial aging) [4]. The strengthening effect of dislocations is amplified through dislocations dissociation which also act as preferential sites for the nucleation of the main strengthening phase T_1_. Multiple investigations of various alloy variations of conventionally produced Al–Cu–Li are already known in the literature [5,6,7,8].

In laser additive manufacturing (AM), such as the laser powder bed fusion (LPBF), due to the high tensile and compressive stresses build-up in the solidifying material because of thermal contraction and expansion, quantitatively high dislocation density is induced similar to conventional cold working [9]. This means, forming T_1_-precipitates through the AM process route is possible without cold working, which is beneficial to shortening the production process. This, however, requires appropriate Cu/Li ratio and aging temperatures along with the presence of high dislocation density which increases the number of nucleation sites for T_1_. Zhang et.al. [10] applied a T6 heat treatment on a LPBF as-built Al–Cu–Li 2195 alloy. They reported a microstructure dominated by T_1_ and a yield strength which was more than double that of the as-built, with a UTS of 247 MPa more. Similarly [11], in an Al–Cu–Li alloy modified with Sc and Zr using the LPBF process, no T_1_ precipitates were reported by the authors in as-built condition but instead Al_3_(Sc,Zr) dispersoids were found. LPBF process feasibility and hot cracking mechanism in as-printed 2195 Al–Cu–Li alloy were also studied by [12,13]. In their earlier work, the current authors [14,15] investigated the LPBF process feasibility and characterization of the microstructure of the AA2099 Al–Cu–Li alloy using a preheated build plate. LPBF preheating not only offers the opportunity to mitigate cracks in crack susceptible alloys, such as 2XXX series Al alloys, but also allows in situ precipitation. The investigations attributed preheating of the build plate during the build job as responsible for hot cracks reduction and also residual stresses that provide the driving force for crack initiation and propagation. T_1_ precipitates were not observed in the as-printed condition, although dislocations were observed. With preheating (in situ) of the build plate, crack-free samples with high relative density and volume fractions of T_1_ (Al_2_CuLi) and T_B_ (Al_7.5_Cu_4_Li) precipitates were characterized.

The heat treatment temperature, whether conventional or preheating, of the AM built part has a significantly different impact on the microstructure and consequently on the mechanical and corrosive properties. Not only do different heat treatments influence microstructural textures but also precipitation, dislocation density and the degree of grain misorientations [16]. For instance, T_1_ volume fraction, length and thickening have all been found to be influenced by aging time and temperature in conventionally manufactured alloys [4,6,8]. Similarly, different conventionally heat-treated Al–Cu–Li alloys have been reported to have different corrosion responses [17,18,19,20,21,22]. These findings indicate that the corrosion behavior of the alloy is as a result of the presence of different types of precipitates in the microstructure. Zhu et al., for example, investigated the electrochemical behavior of precipitates present in the Al–Cu–Li alloy system [21,23]. The authors reported that the precipitate rich in Cu, Fe, or Mn were more cathodic to the matrix and among the numerous Cu and Li-containing precipitate, Al_7.5_Cu_4_Li (T_B_) [23]. The authors further asserted that once most of the Li was lost due to selective dissolution, leaving behind Cu-rich precipitate, these precipitates became progressively cathodic. On the other hand, Li and Cu-bearing precipitates, such as Al_2_CuLi (T_1_) and Al_6_CuLi_3_ (T_2_), were reported to have lower potential than the matrix [23]. As a result, they are both expected to dissolve more easily than the matrix [21]. Ma et al. concluded that T8 conditions led to more severe localized corrosion susceptibility than T6 or T3 due to localized plastic deformation during pre-age cold working and heterogeneous precipitation of T_1_ phase during subsequent artificial aging [24]. Grains with a high Schmidt factor tends to result in localized corrosion as T_1_ precipitation is favored in these areas [24]. In a separate study, Ma et. al. argued that high Cu-containing precipitates were more electrochemically active than low Cu-containing precipitates due to the relatively high level of lithium present in the former particle [19].

In additively built 2XXX series alloys, O. Gharbi et. al. investigated the electrochemical properties [25]. Their work concluded that AM 2024 had a lower dissolution rate than AA 2024 due to the lack of S phase (Al_2_CuMg) and micrometer-sized particles in AM2024 [25].

In this work, in situ preheating influence on the evolution of precipitation microstructure, mechanical and corrosive properties of LPBF-printed Al–Cu–Li alloy (AA2099) has been investigated and compared with conventional AA2099-T83 alloy. The purpose is to study how preheating influences the above-mentioned properties compared to the same alloy when processed and heat-treated using conventional means, such as T83.

## 2. Materials and Methods

The powder used in this work was obtained from gas atomization by Nanoval GmbH, Berlin, after the ingot was cast using a vacuum induction melting oven at the RWTH-Aachen University. An AA 2099-T83 175 × 63 × 175 mm^3^ metal sheet also used in this work was purchased from Smith Metals UK. The chemical composition of the gas atomized powder determined with the inductively coupled plasma optical emission spectroscopy (ICP-OES) and that of 2099-T83 as specified by the supplier are both listed in Table 1. The as-received powder was characterized using the CAMSIZER X2 (Retsch Technologies GmbH) in terms of particle size/distribution and the flowability (Hausner’s ratio) was determined with the help of a STAV II Jolting volumeter (J. Engelsmann AG) according to DIN EN ISO 3953:2011-05 standards. The flowability was determined from calculated Hausner ratio where three averaged tapped densities (1.68 g/cm^3^) were divided by three averaged apparent densities (1.37 g/cm^3^) to yield a Hausner ratio of 1.23. According to the Hausner flowability index, this ratio can be described as fair. A median particle diameter (d50) of 38.3 µm was recorded, with a somewhat good sphericity (SPHT3) of 0.782 as well as a high aspect ratio (b/l3) and symmetry (Symm3) of 0.816 and 0.908, respectively. The powder was always dried ahead of printing in a VT6025 vacuum drying oven, Thermofisher Scientific GmbH oven, at 90 °C for at least six (6) hours.

All the LPBF print jobs were carried out with the Aconity Mini printer (IPG Photonics YLR 400, F-Theta lens (f = 420 mm, and λ = 1030–1080 nm) using a spot diameter of 80 µm. The print jobs were carried out under Ar atmosphere (<2000 ppm O_2_) with a fume extraction velocity of 1.2 m/s. Print geometries were designed and sliced using the AUTODESK NETFABB PREMIUM 2022 software. A simple hatch scanning strategy was used where the scan vectors rotated 90 °C about each subsequent layer. Printed cuboid geometries (5 × 5 × 10 mm^3^) and cylindrical samples (Height—45 × diameter—8 mm) which were machined according to DIN 50125 B4 specifications were built along the Z-axis to the platform. Tensile test was conducted with the INSTRON 8033 tensile test equipment using a crosshead speed of 0.17 mm/min at room temperature for five test samples of each of the investigated alloys according to ISO 6892-1:2019(E) standard. The LPBF cuboid samples were built in as-built (room temperature) and preheated conditions using the temperatures 440 °C, 460 °C, 480 °C, 500 °C and 520 °C. The temperatures were chosen based on previous experimental and phase simulation investigations by the current authors which indicated the formation temperature of T_1_ from the melt at ~520 °C and subsequently with T_B_ at ~490 °C until complete solidification. For each preheating temperature, optimized process parametric samples (Table S1) were chosen for further analytics (not less than 99.0% relative density (RD)). The highest RD of 99.7% among all the printed preheating samples was however achieved at 500 °C and thus 500 °C was chosen as the build temperature for tensile test and for APT investigations. Unless stated otherwise, all microstructure characterizations of the LPBF-built samples were carried out at the middle portions of the samples.

After microstructure and mechanical property (hardness) correlational analysis, three preheating temperatures (480 °C, 500 °C and 520 °C) were selected for electrochemical investigations alongside the conventional T83 sample. Samples for electrochemical characterization were cold-mounted in epoxy, mechanically ground under ethanol to 4000 grit finish and dried in warm air. Epoxy glue was applied on the samples to prevent crevice effect during the measurements. The electrochemical measurement was performed using Gamry 600. The setup used saturated calomel electrode and a platinum wire counter electrode. Electrochemical testing, including the measurement open circuit, potentiodynamic polarization and electrochemical impedance spectroscopy (EIS) were performed under 0.1 M NaCl. The open circuit measurement was measured for 3600 s. The potentiodynamic measurement parameters was 0.5 mV/s for the scan rate with commencing from −200 mV to +200 mV from open-circuit potential (OCP). EIS was also measured at the open-circuit potential. The impedance measurements were conducted over a frequency range of 10 kHz down to 10^−2^ Hz using a 10 mV amplitude of sinusoidal voltage in a Faraday cage to minimize external interferences.

The Zeiss light optical microscope (LOM) and scanning electron microscope (SEM); (Field Emission Gun (FEG), Oxford Instrument Inca X-sight Energy Dispersive Spectroscopy (EDS)/Electron Backscatter Diffraction (EBSD) detectors) were used for microstructure investigations. Relative densities of the built samples from light optical micrographs were quantified using the FIJI IMAGE J software. Etching was performed with 0.5% hydrofluoric acid (HF) for 60 s. The Matlab MTex toolbox was used to analyze all the EBSD characterized samples. Kernel average misorientation (KAM) and geometrically necessary dislocations (GND) maps were also generated using the MTEX toolbox. Angular misorientations measured between two neighboring grains were set as high angle if the angle θ>10°. Three-dimensional (3D) nano-characterization was performed on the commercially obtained AA2099-T83 as well as the preheated AM-built samples (500 °C) using a Local Electron Atom Probe (LEAP) 4000× HR system (CAMECA Instrument Inc., Fitchburg, WI, USA). Voltage-pulse mode (frequency of 125 kHz, pulse fraction of 20%, and detection rate of 0.5%) was selected for data collection. All measurements were conducted at the base temperature of 30 K. The APT samples (tips), which had radii of about 30 nm to ensure good evaporation, were fabricated using the easy-lift method and subsequent annular milling via the FEI Helios Nanolab 660 SEM-focused ion beam (FIB) system. The collected data were reconstructed and analyzed using IVAS module in AP suite 6 software (CAMECA Instrument Inc., Fitchburg, WI, USA).

## 3. Results

### 3.1. LPBF Microstructure—As-Built Cuboid

**Figure 1:**
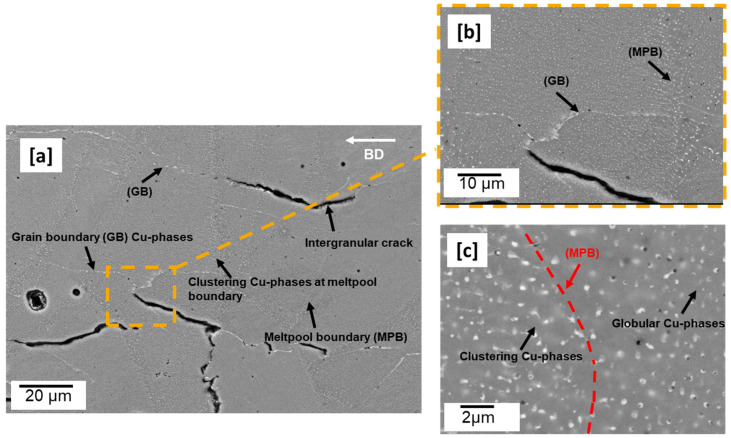
Microstructure of as-built sample (**a**) showing predominantly intergranular cracks (**b**,**c**) clustering of Cu-rich phases both at the melt pool and grain boundary regions (see Figure S1).

Figure 1 shows the scanning electron micrographs (SEM) of an as-built microstructure. Two main defects can be observed: gas pores and cracks. The cracks are mostly intergranular, propagating predominantly along the building direction (BD), (Figure 1a). Globular Cu-rich phases (appear whitish) are seen all over the microstructure and appear to be more clustered at the melt pool boundary regions (Figure 1a,b). These phenomena were also similarly observed by [12] in LPBF-built 2195 Al–Cu–Li alloy. 2XXX series, including Al–Cu–Li alloys have large solidification intervals (~180 °C for this alloy) and are susceptible to hot cracks [26]. When processed with LPBF, which inherently has high cooling and solidification rates from µ-sized melt-pools with short lifetime, slowing down the cooling and solidification rates can inhibit cracks at the last stage of solidification [12]. The use of a preheated build plate during the building process slows down the solidification and cooling rate because it prolongs the melt pool lifetime and decreases the temperature gradient.

**Figure 2:**
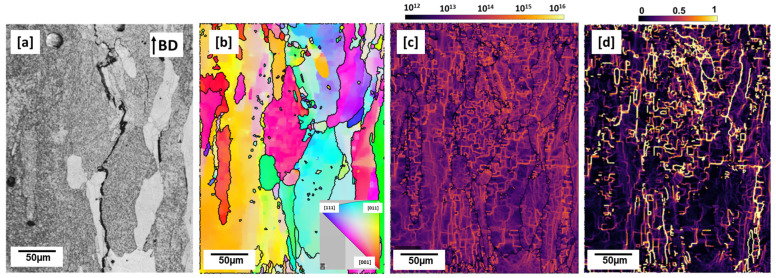
As-built cuboid (**a**) band contrast image (**b**) inverse pole figure (IPF) map (**c**) geometrically necessary dislocation (GND) map, dislocation density: 3.8949 × 10^13^ m^−2^ (**d**) kernel average misorientation (KAM) map: 0–1.

From the as-built micrograph (Figure 1a–c), the observed Cu-rich phases appear to be more enriched at the melt pool and grain boundaries regions than in the matrix. The Cu diffusivity at the melt pool boundary region is aided by the localized thermal cyclic reheating and sometimes remelting from underlying substrate layers and overlapping scan tracks [27]. According to [27], track–track overlap and reheating/remelting from underlying substrate layer offer an alternative diffusional path for Cu, such as seen at areas around melt pool boundaries (Figure 1b,c). In the band contrast image (Figure 2a), propagated crack along the building direction and grain boundaries is clearly shown. The grains are typically coarse and columnar, and parallel along the build direction (Figure 2b). The width of the columns is observed to be between ~25 and 50 µm and as long as between ~100 and 250 µm. The main driver of coarse columnar grains is the inherently high temperature gradient as the heat flux is conducted away from the substrate plate, typically known to be in the region of 10^6^ K/m for the LPBF process [28]. These grains, apart from being an inherent source of anisotropy, unlike equiaxed grains, are poor at accommodating strain along their axes and relief stresses effectively [28]. The columnar grains have a high aspect ratio of 3.12.

High thermal stresses induce a high dislocation density in laser-based additively manufactured parts [9]. Plastic deformation in a polycrystalline material due to imparted strains consequently results not only in the formation of dislocations, but also their movements, annihilation and storage [29]. According to [29], at the mesoscopic length scale in polycrystalline materials, grains with different orientations have intergranular variances in response to plastic deformation according to Ashby’s single slip model. They further asserted that for microstructure continuity, intragranular deformation must also take place to avoid voids. In order to maintain strain compatibility, therefore, microstructures store some dislocations as geometrically necessary dislocations (GNDs). GNDs have a net-non zero Burgers vector, having a resultant geometric effect on the lattice (lattice curvature) [29]. The authors [30,31] related the dislocation density tensor to the elastic strain fields and the curvature of a crystalline lattice.

From electron back scatter diffraction (EBSD) measurements, therefore, crystallographic misorientations between neighboring crystals due to lattice curvature can be observed in misorientation maps and the amount of GNDs quantified [29]. Figure 2c shows a GND map of the as-built microstructure with an average dislocation density of 3.8949 × 10^13^ m^−2^, which is higher compared to the 2.13 × 10^13^ m^−2^ reported by the authors of [10] in an as-built 2195 sample. The dislocation line defects, as seen in Figure 2c, appear to be less dense at the bottom of the sample and denser as it approaches the top of the sample. Heat accumulation during the print process is expected to be higher in the bottom of the samples closer to the build substrate and may have led to some dislocation recovery. Kernel average misorientation (KAM), which is a measure of the average misorientation between a kernel point and its neighboring points not including grain boundaries, can be used to determine the degree of local grain misorientation or lattice curvature as well as dislocation density, such as GNDs [32,33]. From the KAM map shown in Figure 2d and as expected, it is evident that the high KAM intensity zones correspond to areas of high dislocation density (Figure 2c). This is so because dislocation accumulation is proportional to the degree of imparted strain.

T_1_ (Al_2_CuLi), which is the main strengthening precipitate of Al–Cu–Li alloys, is widely believed to nucleate at dissociated dislocation sites where dislocations split into Schockley partials [34]. In spite of the high dislocation density, no T_1_ plate precipitates are observed in the as-built microstructure (Figure 1 and Figure 2), consistent with the authors earlier reporting that time and temperature are significant factors for T_1_ precipitation in AM-built samples [15]. This means that even at the nucleation temperature, T_1_ precipitation is kinetically inhibited because of insufficient time for diffusion.

### 3.2. LPBF Microstructure—Preheated Cuboid (440 °C, 460 °C, 480 °C, 500 °C, and 520 °C)

Although the as-built microstructure shows a high dislocation density, which suggests that the conventional requirement for cold working can be avoided, it is clear, based on the characterized as-built microstructure, that appropriate heat treatment cannot be avoided in order to facilitate T_1_ precipitation. As such, authors of [10,11], after performing T6 heat treatment (solutionized, 520 °C for 1 h, water quenched and artificially aged at 170 °C for 6 h) on as-built samples, found T_1_. However, to have a competitive edge over conventional manufacturing, the reduction in post-production steps, such as T6 heat treatment will be economically beneficial in the qualification of Al–Li alloys for AM processes. The evolution of precipitation microstructures in preheated LPBF-built AA2099 alloy samples has been investigated at the following preheating temperatures: 440 °C, 460 °C, 480 °C, 500 °C and 520 °C. The AM microstructures, mechanical and corrosive properties have been compared with the same alloy but conventionally processed and heat-treated (T83) and the findings discussed.

**Figure 3:**
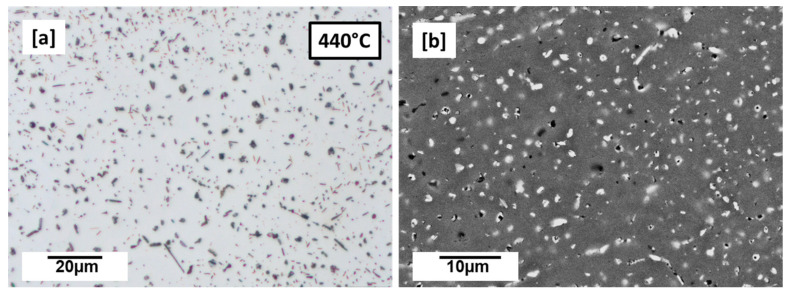
440 °C (**a**) etched (0.5% hydrofluoric acid (HF), 60 s) LOM preheated-built samples (1000×) °C showing globular Cu-rich phases and plate-like precipitations (**b**) SEM-BSE (5000×) micrograph of 440 °C.

The 440 °C preheated microstructures are shown in Figure 3a,b. The microstructure is clearly seen to predominantly have many globular Cu-rich phases which appear as dark-grey spots (Figure 3a) or whitish (Figure 3b). Along with the globular Cu-rich phases, the preheated (440 °C) microstructure also shows very small plate-like and rod-like precipitations (Figure 3a). The plate-like and rod-like precipitates are characteristic of the T_1_ and T_B_ precipitates, respectively, which were earlier characterized by the authors using synchrotron high energy X-ray diffraction (SHE-XRD) and phase simulation as the main precipitates when this alloy composition is processed under preheating conditions [14,15].

**Figure 4:**
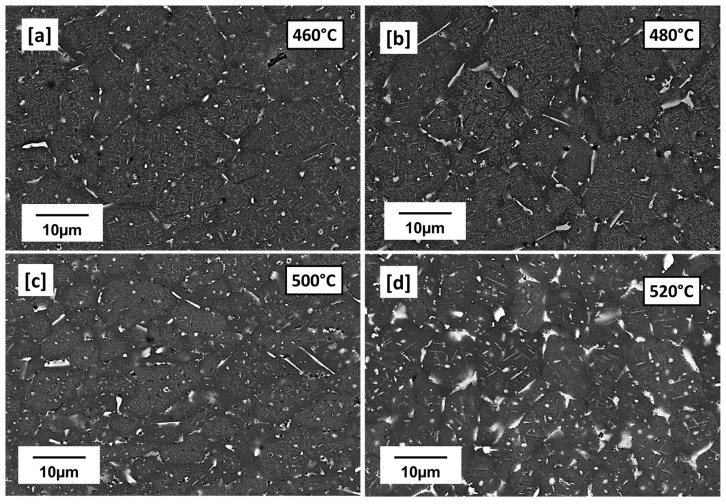
Preheated-built samples, SEM (5000X); (**a**): 460 °C; (**b**) 480 °C, (**c**) 500 °C, (**d**) 520 °C: showing increasing low melting Cu-rich phases (See Figure S1) and PFZ with increasing preheating temperature.

**Figure 5:**
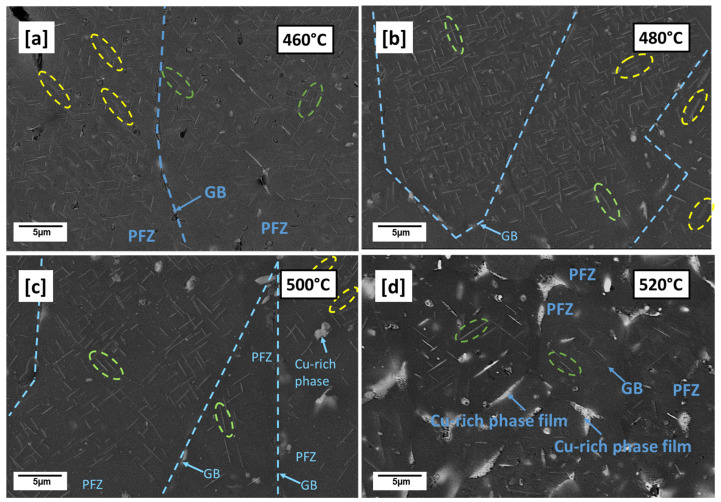
Preheated-built samples, SEM (10,000×); (**a**): 460 °C; (**b**) 480 °C, (**c**) 500 °C, (**d**) 520 °C: showing the influence of increasing preheating temperature on precipitate morphology; yellow mark—T_B_, green mark—T_1_.

With further increase in preheating temperature from 460 °C to 520 °C (Figure 4a,d), the microstructures appear differently from 440 °C (Figure 3a,b). Many intragranular globular Cu-rich phases observed at 440 °C (Figure 3a,b) can be seen to have reduced while thick Cu-rich phase films can be mostly observed on the grain boundaries as preheating temperature increases to 520 °C. Grain boundaries serve as sinks for solute atoms and vacancies during solidification; preheating, which slows down solidification rate therefore favored the diffusion and segregation of solute Cu atoms to the grain boundaries forming these low melting Cu-rich phases and depleting surrounding zones off Cu, available for precipitation [15]. PFZ areas are therefore observed mostly around grain boundaries. The appearance of both plate-like and rod-like precipitates suspected to be T_1_ and T_B_ precipitates, respectively, can also be observed as the preheating temperature increases to 520 °C (Figure 5a,d). The increasing preheating temperature clearly influences the particle density and the plate length. The plate lengths even for the same preheating temperatures are observed not to be the same due to the resultant effect of the highly localized thermal inhomogeneities of the LPBF process. The plate-like particle density can be observed to increase from 460 to 480 °C and becomes less dense but coarser between 500 and 520 °C. Between 500 and 520 °C, is also the prevalence of more PFZ and Cu-rich phases. In general, however, time and temperature can be said to have allowed more precipitate growth, coarsening and dissolution of some amidst the formation of more PFZ and Cu-rich phases. Mean T_1_ plate length of ~1.096 µm, ~1.225 µm, ~1.415 µm, and ~1.78 µm were respectively recorded for 460 °C, 480 °C, 500 °C and 520 °C preheating temperatures. A tendency of increasing mean plate length with preheating temperature can be observed. The measured mean length determined in the preheated LPBF-built samples by far exceeds the reported values for conventional alloys which ranges between 40 and ~160 nm by [35], 40 and 55 nm by [4] and up to about 200 nm by [7], all under different conventional post heat treatment temperatures.

The magnifications used in this study can be described as insufficient to observe any significant increase in thickness of the characterized plates resulting from the preheating effect. In conventionally processed alloys, thickness of up to 2 nm have been reported [4,36]. A stable T_1_ plate thickness is beneficial, as increasing thickness over long term aging reportedly decreases yield strength [4].

In the conventional process, a variety of T_1_ microstructures are attainable through the variation of factors, such as the degree of prior deformation, heat treatment temperatures and their duration [4]. These factors affect the amount of dislocations and therefore the number of dislocation sites, particle density, and volume fraction and in the case of T_1_ plate-like precipitates, plate thickness and length. More specific to AM, the residual and thermal stress build-up, highly localized non-equilibrium solidification and cooling conditions, non-uniform temperature distribution as well as the cyclic reheating of already solidified layers by underlying substrate and overlapping layers further contribute to the unique AM microstructures, as shown in Figure 4 and Figure 5.

**Figure 6:**
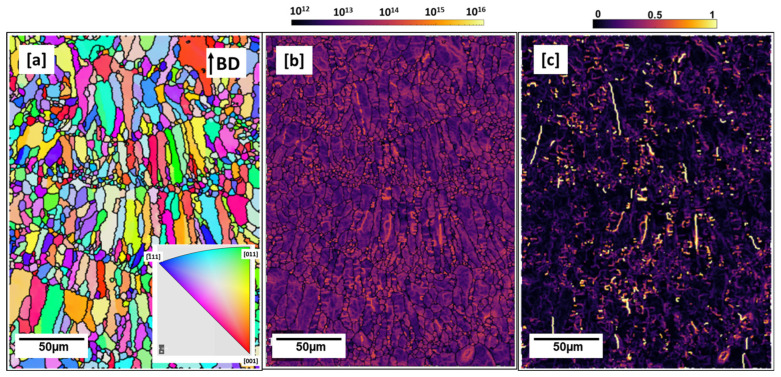
Preheated 500 °C cuboid (**a**) inverse pole figure (IPF) map (**b**) geometrically necessary dislocation (GND) Map; dislocation density: 2.2 × 10^13^ m^−2^ (**c**) kernel average misorientation (KAM) Map: 0–1.

The IPF map of the 500 °C preheated microstructure is shown in Figure 6a. Coarse and shorter columnar grains (width of columns not more than 20 µm and length not more than ~60 µm) compared to the as-built microstructure (Figure 2b) can be seen to have grown from the melt pool boundary, parallel to the building direction. The observed grain growth behavior is not uncommon in additively built microstructures [37]. The columnar grains are truncated in the upper part by equiaxed grains at the melt pool boundary. The temperature gradient (G) to rate of solidification (R) typically governs solidification grain morphology in the µ-sized melt pools. G/R is typically highest at the bottom of the melt pool and as a result falls in the region of columnar-dendritic solidification as equiaxed solidification conditions are satisfied at the upper part of the melt pool where temperature gradient is lower [38].

Compared to the as-built condition (Figure 2a,b), which shows coarse columnar grains due to the very high temperature gradient along the building direction, the heating plate effect (500 °C) can be described as having facilitated the lowering of the temperature gradient, hence, the shorter observed columns. No clear texture can be observed. The grains aspect ratio of 2.03 was calculated from MTEX. From the GND map (Figure 6b), calculated GND density recorded a lower dislocation density of 2.2 × 10^13^ m^−2^ compared to the as-built condition which can be attributed to dislocation recovery due to the high heat treatment temperature exposure. The intensity of misorientation between neighboring grains, as shown with the KAM map (Figure 6c), is also observed to be lower due to the comparatively lessened temperature gradient as a result of the preheating effect.

### 3.3. Conventional Microstructure—T83 Alloy

**Figure 7:**
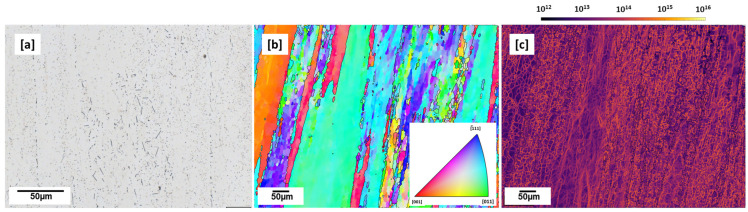
T83 (**a**) etched micrograph showing rod and plate-like precipitates (**b**) IPF (Y) map showing large grains in rolling direction (**c**) GND map with high dislocation density (6.612 × 10^13^ m^−2^), 3% cold worked.

The microstructure of commercially obtained T83 AA2099 alloy is shown in Figure 7a–c below. The alloy was solution heat-treated, quenched, 3% cold worked, and artificial aged at 150 °C [18]. Figure 7a shows several many small globular, plate-like and rod-like precipitates which vary in lengths. The IPF Map shows, in Figure 7, long elongated grains along the rolling direction. Average GNDs of 6.612 × 10^13^ m^−2^ was computed with MTEX for the T83 sample (Figure 7c). This is marginally higher than the average GNDs computed in as-built (3.8949 × 10^13^ m^−2^) and under 500 °C preheated (2.2 × 10^13^ m^−2^) conditions. This may be due to the 3% pre-aging deformation and the relatively lower aging treatment temperature of 150 °C [18] which was performed on this alloy compared to the 500 °C preheating temperature. The microstructures of the T83 and AM sample (500 °C preheated) are further characterized with APT and discussed below.

### 3.4. Atom Probe Tomography Characterization of T83, Preheated 500 °C

**Figure 8:**
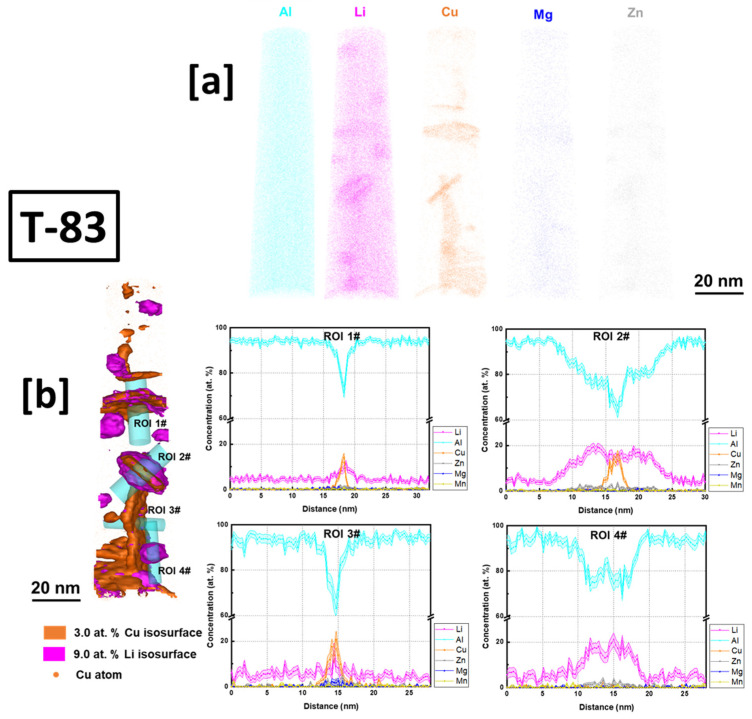
APT (**a**) tomography maps of Al, Li, Cu, Mg and Zn, (**b**) isoconcentration surface and proxigram of T83 conventionally obtained alloy AA 2099.

**Figure 9:**
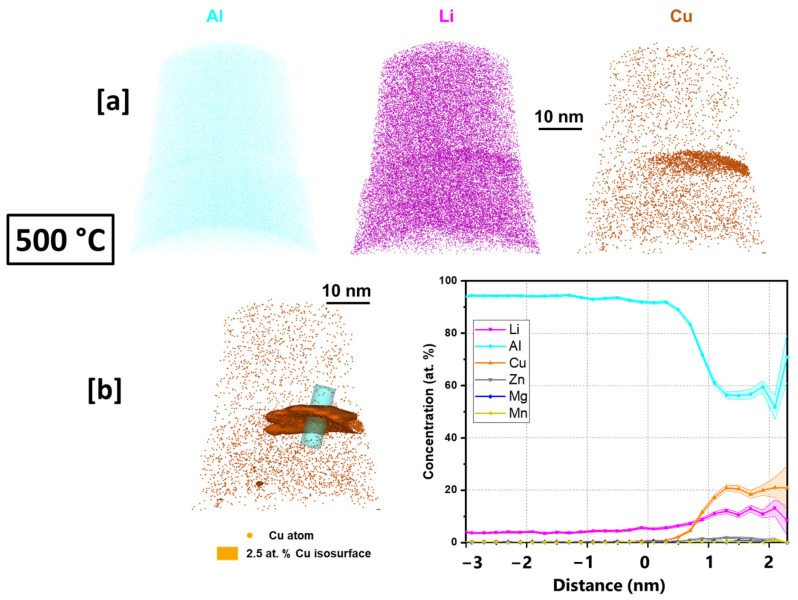
APT (**a**) tomography maps of Al, Li, and Cu (**b**) isoconcentration surface and proxigram of 500 °C preheated sample.

The constituent elements and their quantitative amounts create competing phases in the multi-phase Al–Cu–Li alloy system. Mg and Zn significantly influence the T_1_ nucleation kinetics [39,40,41]. Mg in such alloys has been found to segregate at the precipitate-matrix interface and also partition within the structure of T_1_ [42,43,44] in conventionally produced Al–Li alloys. The stoichiometric composition of T_1_ as well as its interface layer with the matrix have been a subject matter of a number of investigations [45]. Atom probe tomography (APT) is beneficial to gain further insight into the elemental composition at the atomic level of precipitates, especially T_1_, its stoichiometry and interface composition with the matrix. Such findings are critical and provides further insight into T_1_ nucleation kinetics. The T_B_ rod-like precipitate has, however, not attracted much research interest as its strength contribution is regarded as minimal [46].

(APT) analysis in this work was conducted on the conventional T83 AA2099 as well as one of the AM-built preheated samples (500 °C). A detection rate of 0.5% was selected for data collection. APT tomography maps, isoconcentration surface from APT data reconstruction and the proximity histograms (proxigrams) as a function of distance from the isoconcentration surface (Figure 8 and Figure 9) of the investigated samples are presented and discussed below.

The conventional T83 alloy shows enrichment of Cu, Li, Mg and Zn in various precipitates at different concentrations in the reconstructed volume maps (Figure 8a). From the region of interest (ROI) 1# and 3#, precipitates enriched with Cu at ~18 at.% and ~20–25 at.%, respectively, and Li atoms at ~14 at.% and ~20 at.%, respectively, can be observed. In ROI 2#, the isoconcentration surface shows a plate-like precipitate encapsulated and shelled by enriched Li atoms. The shell has Li enriched at ~20 at.% compared to the inner core plate with ~18 at.% Li. In ROI 4#, the spherical precipitate is almost dominated by only Al and Li atoms with little or no Cu atoms concentrated in the precipitate. Li is enriched up to about ~30 at.%. From the isoconcentration and proxigram analysis, ROI 4# is characteristic of the spherical Al_3_Li (δ) precipitate and the same in ROI 2# can be described as encapsulating a T_1_ plate. δ encapsulation or shelling of intermetallic particles (Al_3_TM –TM: transitional metals) is not uncommon in the Al–Li alloy system as shelling of Al_3_Zr by δ have been equally reported [47]. ROI 3#, in particular, on the bases of the atomic concentration of Cu and Li, progresses towards a T_1_ precipitate but with slight difference in stoichiometry. Deviations in stoichiometric composition of T_1_ in conventional Al–Cu–Li alloys have been previously explained [42,44,45]. They were attributed to instability in ionization behavior of atoms in and around the T_1_ plates or matrix concentration contortion with precipitates due to aberrations during the APT analyses. From ROI 3#, significant enrichment of Mg atoms in the T_1_ plate can be observed, consistent with findings by [44,45] as opposed to findings by [42].

In the preheated sample (Figure 9a), the enrichment of both Cu and Li atoms in the precipitate can be observed. The proxigram of the analyzed precipitate shows enrichment of Cu and Li atoms of ~22 at.% and ~18 at.%, respectively, which progresses towards T_1_ precipitate but with deviation (T_1_ nominal: 25 at.% Cu, and 25 at.% Li.) in stoichiometry (Figure 9b). Some Mg atoms can be observed in the precipitate with no clear segregation of Mg between the matrix and the precipitate interface. In both the conventional T83 as well as the additively manufactured samples, Mg atoms within the T_1_ structure are observed. This further supports the findings by [44] concerning the aiding of T_1_ nucleation by Mg.

### 3.5. Mechanical Properties—Hardness, and Tensile properties

**Figure 10:**
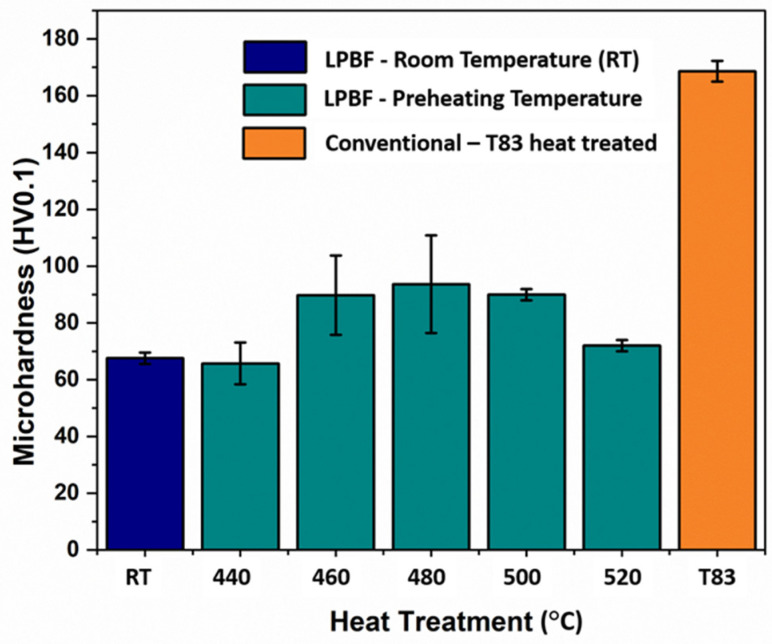
Microhardness comparison of room temperature (RT) [15], preheating (460 °C, 460 °C, 480 °C, 500 °C, and 520 °C [14]) and commercially obtained conventional T83 samples.

Microhardness (HV0.1) measured from the preheated samples (440 °C, 460 °C, 480 °C, 500 °C, and 520 °C) is compared with room temperature (as-built) and conventional T83 Al–Cu–Li alloy, as shown in Figure 10. From the preheated samples, the 440 °C recorded the least microhardness of 65.6 HV0.1, slightly lower than as-built 67.6 HV0.1 whose strength can largely be attributed to strength contribution from internal residual stress [48]. From the observed 440 °C microstructure in Figure 4a,b, the hardness can be attributed to the precipitation hardening effect of the many small globular precipitates. While the hardness between 460 °C (89.8 HV0.1) and 500 °C (89.9 HV0.1) preheated samples are nearly the same, 480 °C increased marginally to (93.6 HV0.1). This may be regarded as the peak hardness under AM preheating conditions, especially because the hardness decreases further to 72 HV0.1 with further increase in preheating to 520 °C (Figure 10). The evolution of the precipitation microstructure with preheating temperatures (Figure 5) can be attributed to the varying hardness properties shown in Figure 10. Of the two dominating precipitates from this alloy composition, T_1_ is known to be the major strength contributor compared to T_B_ [15]. However, T_1_ precipitation strengthening effect is equally governed not only by the volume fraction of the precipitates but also the structure of the precipitates, the size, distribution as well as the aspect ratio [6]. The aging temperatures and times influence the aspect ratio of the plate precipitates and consequently their strengthening effect [6]. From the micrographs in Figure 5, variances can be observed in terms of particle density and mean plate length with the increase in preheating temperature. The higher particle density of T_1_ plates can be attributed to the hardness increase between the 460 °C, 480 °C and 500 °C as opposed to as-built (Figure 1a–c), 440 °C (Figure 3a,b) and 520 °C (Figure 5d). Highest particle density of the fine plate-like precipitates can be observed at 480 °C (Figure 5b), which may be the reason behind the peak hardness. In addition to the lower particle density, the drop in hardness at 500 °C and 520 °C from the peak hardness temperature may be attributed to slight coarsening of the T_1_ plates in terms of plate length and the probable loss of their semi-coherency as well as the presence of more PFZ and the build of Cu-rich phases. The preheating temperature also influences the dislocation density and consequently the dislocation strengthening effect due to the critical plate size.

Compared to the conventional T83 sample with hardness of 168.6 HV0.1, the preheated samples were of inferior hardness. This can be attributed mainly to the precipitation strengthening contribution of the presence of precipitates, such as T_1_, T_B_ and the coherent Al_3_Li spherical precipitates as characterized by the APT in Figure 8.

**Figure 11:**
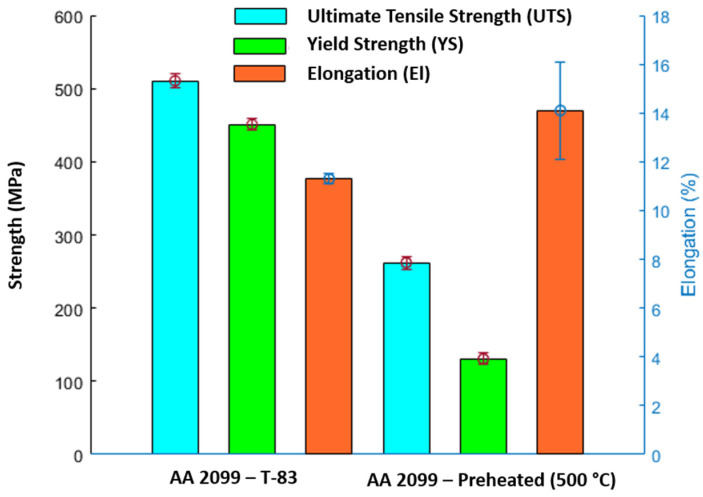
Tensile strength: comparison between 500 °C, and T83 heat-treated conventional AA2099 Al–Cu–Li alloy.

A plot of averaged tensile properties (stress–strain curve), determined for both preheated (500 °C) and T83 heat-treated tensile samples (B4), is shown in Figure 11. The conventional T83 sample shows better yield and ultimate tensile strength properties but falls short in elongation compared to the additively built sample. Yield strength (YS) of 451 MPa (RP0.2), ultimate tensile strength (UTS) of 511 MPa and elongation (El) of 11.3% were recorded for the conventional T83 alloy. Similarly, YS (RP0.2) of 131 MPa, UTS of 262 MPa and elongation of 14.1% were recorded for the additively built sample. The higher strength properties in the conventional alloy can be attributed mainly to precipitation strengthening contributions from the multiple precipitates as already characterized. In the preheated sample at 500 °C, the authors reported that it was dominated by mainly T_1_ and T_B_ plates (more volume fraction of T_1_) [15]. Since the T_B_ strengthening effect is regarded as minimal [46], precipitation strengthening contribution can be attributed to the T_1_ plates. Plates, such as T_1,_ with prolong aging temperatures and time, can lead to YS and UTS decrease [4,49]. It therefore stands to reason that as it was characterized both in Figure 5 and Figure 10, at 500 °C, the lengthening of the plates and the high aspect ratio thereof might have led to a loss of semi-coherency and as such could not have the maximum strength contribution effect. The strength contributing effect of the different types of precipitates with regards to their size and distribution under AM process need to be defined for optimization of the microstructure.

### 3.6. Corrosion Properties—T83 (Preheated: 480 °C, 500 °C, and 520 °C)

**Figure 12:**
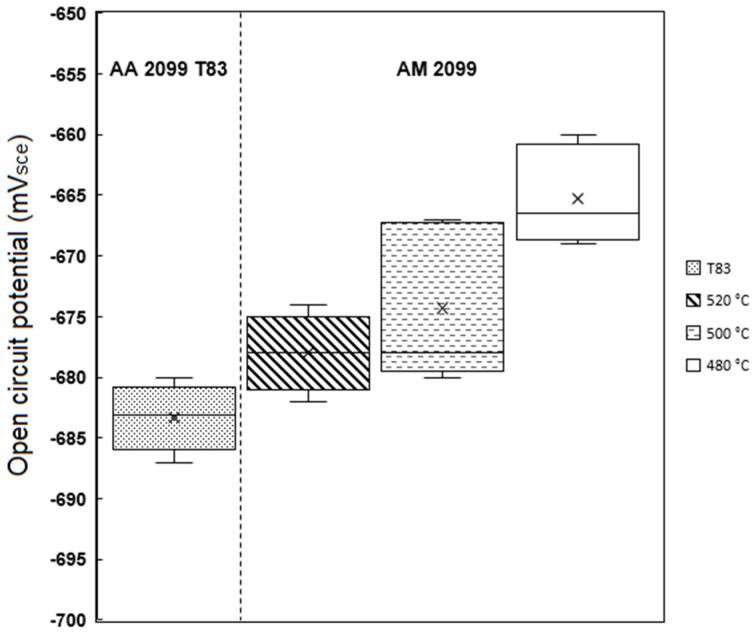
Open circuit potential (OCP) of AM-preheated and conventional T83 Al–Cu–Li alloys compared.

The AM alloy samples which exhibited decreasing hardness (Figure 10) were selected for characterization of their electrochemical behavior as the preheating temperatures (480 °C, 500 °C, and 520 °C) increased. Additionally, the electrochemical behavior of the conventional T83 sample was compared with that of the AM-produced alloy. Figure 12 illustrates the open-circuit potential (OCP) of both the preheated AM samples and the conventional AA 2099-T83 sample after reaching the steady state condition in a 0.1 M NaCl solution for 1 h. The OCP of the preheated AM alloy showed a slight decrease from −665 mV for the 480 °C sample to −678 mV for the 520 °C sample. The microstructures shown in Figure 4 indicate the formation of coarse, low-melting Cu-rich phases in the grain boundary at temperatures above 500 °C, which correlates with the decrease in open-circuit potential as the preheating temperature increased. The precipitation of Cu from the matrix reduces the matrix’s potential, consequently affecting the overall potential of the sample, which aligns with previous investigations [50,51,52]. In comparison to the T83 sample, all the AM-printed samples exhibited a more positive potential, which can be attributed to the observed precipitation behavior. As mentioned earlier, the average size of plate-like and rod-like precipitates in the T83 sample is much larger (5 µm) than those observed in the preheated AM ones, which range from 2 to 5 µm. The APT results shown in Figure 9 reveal significant Cu enrichment in the rod or plate precipitates. Consequently, the larger the precipitate size, the more copper it consumes from the matrix, leading to a more negative potential for AA 2099.

**Figure 13:**
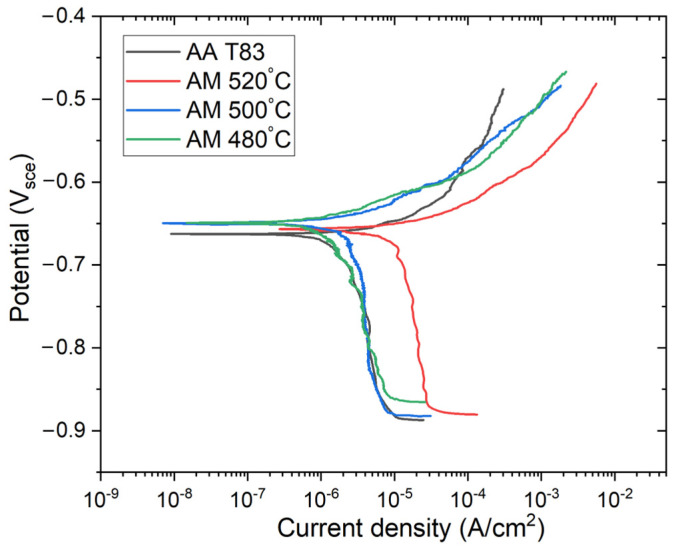
Potentiodynamic polarization of AM-preheated and conventional T83 Al–Cu–Li alloys compared.

The corrosion behavior was characterized using potentiodynamic polarization. As depicted in Figure 13, both the T83 and AM samples did not exhibit a passive region or obvious pitting potential. This suggests that local corrosion had already occurred at the corrosion potential (Ecorr). In this study, corrosion current densities (I_corr_) were calculated by performing Tafel analysis on the anodic branch, as the cathodic branch displayed diffusion-controlled behavior. The anodic Tafel slope showed a slight increase due to the rising preheating temperature. Furthermore, the cathodic branch also shifted towards higher values as the preheating temperature increased. The shifting of the cathodic branch indicated that the cathodic reaction was affected by the preheating temperatures. Hence, the microstructural evolution obviously contributed to the corrosion behavior in the AM Al–Cu–Li samples.

From Table 2, the heat treatment temperature can be seen to influence the corrosion current density (Icorr) of the AM samples. The average corrosion current density was estimated to be 1.3 µA/cm^2^, 5.1 µA/cm^2^, and 10.3 µA/cm^2^ for 480 °C, 500 °C, and 520 °C, respectively. In contrast, the corrosion current density of the T83 sample is only 0.97 µA/cm^2^, making it the lowest of all the samples. This indicates that the T83 sample had a slower corrosion rate compared to the AM samples. The closest AM sample to the T83 in terms of the corrosion current density was the preheated 480 °C sample.

Variations in corrosion resistance can be attributed to the different microstructures resulting from the different heat treatment exposures to which both the AM (Figure 4 and Figure 5) and the T83 samples (Figure 7) were subjected. During the layer-wise build-up of the LPBF process, as a result of the high solidification and cooling rates as well as steep temperature gradient in the micro-sized melt pools, diffusion is typically limited and solute trapping at the solidification front tends to occur [53,54]. Engaging a heated substrate plate therefore lowers temperature gradient and slows down solidification, thereby allowing sufficient time and temperature for phase transformations. During the layer-wise build-up also, underlying substrate layers are exposed to multiple heating cycles and in some cases partial remelting, with the potential of triggering in situ precipitations [55]. This means that nucleation from the melt during solidification as well as solid state precipitations are both likely to have occurred in the AM-produced alloy, shown in Figure 5. The chosen preheating temperatures and the inherent localized thermal inhomogeneities of the AM process as described above created the conditions for the types of precipitations formed, such as T_1_ and T_B_ and their variances in distribution and sizes. The formation of the T_1_ and T_B_ phases will consume a certain amount of copper from the matrix, so that the potential difference between T_1_, T_B_ and the matrix will be smaller [20,51].

At a preheating temperature of 480 °C, the formation of Cu-rich phases occurs at a slower rate compared to 500 °C or 520 °C, resulting in limited formation of the precipitation-free zone (PFZ). The presence of a smaller PFZ, discontinuous Cu-rich phases at the grain boundaries, and better distribution of T_1_ and T_B_ contribute to increased corrosion resistance, as observed in the preheated 480 °C sample. This finding is consistent with the results reported by X. Wang et al. and X. Lei et al., which showed that the precipitation of discontinuous Cu-rich phases at the grain boundaries up to a certain morphology, can elevate the grain boundary potential closer to or higher than that of the matrix potential, thus preventing intergranular corrosion [20,56].

As the preheating temperature increases to 500 °C, more Cu is segregated to the grain boundaries, resulting in the formation of a Cu-rich phase films. This leads to the consequent consumption of Cu from the matrix, causing the precipitation-free zone (PFZ) to widen [20,51]. This microstructural condition is assumed to result in a significant increase in Icorr, reaching 5.11 µA/cm^2^. The situation worsens further at 520 °C, as more coarse, low-melting Cu-rich phase films form at the grain boundaries, accompanied by a widening PFZ. Consequently, there is a significant loss in corrosion resistance, as indicated by a higher Icorr. The PFZ, as described in various literature, is a region with a lower Cu concentration than the surrounding areas, making it considerably more anodic and susceptible to intergranular corrosion [51]. The formation of low melting Cu-rich phase films as well as Cu-containing precipitates, such as T_1_ and T_B_, can be ascribed as the main reasons why the cathodic branch shifts towards a higher value since these precipitates serve as the location where the cathodic reaction occurs.

When comparing the T83 and AM samples, the T83 sample is expected to have fewer Cu-rich precipitates and a smaller PFZ due to the nature of its process. The T83 sample undergoes a post-heat treatment process, which means it does not directly undergo the solidification process as the AM sample does during the preheat treatment. Furthermore, the aging temperature for the T83 sample is typically lower than the temperature used in preheating the AM samples. This limits the possibility of Cu diffusion and prevents the formation of coarse Cu-rich precipitates observed in the AM samples. Additionally, the T83 sample undergoes pre-aging cold working of 3% after solution annealing and quenching. This artificial aging process allows a better distribution of solid-state precipitations at dissociated dislocation sites, which promoted the nucleation of T_1_ in the matrix and suppressed the grain boundary precipitation [24].

**Figure 14:**
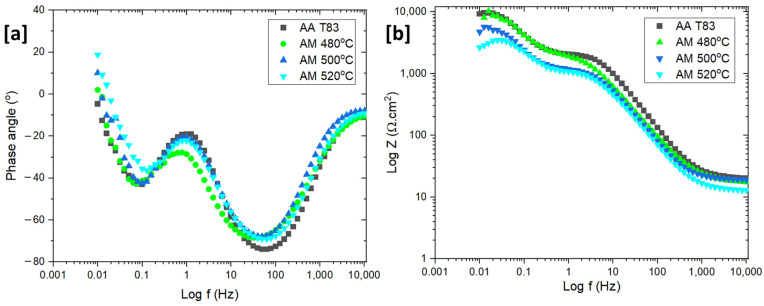
Bode plots of AM alloy and conventional T83 sample in NaCl: (**a**) Bode plot (phase angle vs. log f) (**b**) Bode modulus plots (log|Z| versus log f).

The Bode plots of the preheated AM and T83 samples are shown in Figure 14. The Bode plots of all the samples reveals similar results. Two time constants are noticeable, the first at a high to medium frequencies (1000 to 1 Hz) which may relate to passivation and the second at a low frequency (0.5–0.05 Hz), indicating activities of the double layer (Figure 14a,b). However, at high frequencies, a rather low polarization resistance was observed, indicating a metastable passivation due to NaCl addition. Aside from the two time constants, the phase angle has a positive value at low frequencies, indicating an inductive loop. However, this inductive loop is only found with the AM samples which is an Indication that the inductive loop could be also affected by porosity on the surface (Figure 14a).

**Figure 15:**
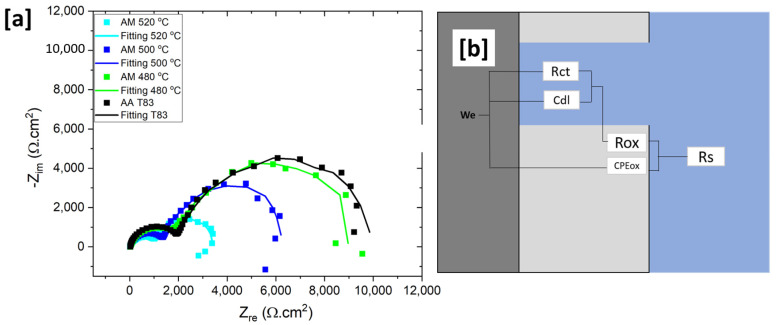
(**a**) Nyquist plot of AM-preheated alloy and conventional sample (**b**) equivalent electrical circuits (EEC) models.

The Nyquist plot shows the typical behavior of the two-layer formation as indicated by the two arc shapes with two different curvatures (Figure 15a). The first arc, which formed during the high frequency scan, is related to oxide resistance (R_ox_) and the second arc to the charge transfer resistance (R_ct_) from the double layer region. The above parameters represent the resistance of each layer towards the charge exchange on the metal surface. As shown in Figure 15a, heat treatment has a substantial impact on the R_ct_ and R_ox_ values. The value of those two types of resistances decreases linearly with increasing heat treatment temperature. The R_ox_ and R_ct_ values at 480 °C are 1.8 kΩ.cm^2^ and 4.2 kΩ.cm^2^, respectively. Then, at 500 °C, both resistances decreased significantly to 1.4 kΩ.cm^2^ and 4.2 kΩ.cm^2^ for R_ox_ and R_ct_, respectively, and went down further at 520 °C. This suggests that charge can move more easily through and out of the metal surface as the preheating temperature increases. Furthermore, the arc of the T83 sample is larger than the AM samples, which is consistent with the potentiodynamic polarization result.

Based on the Nyquist and Bode plots (Figure 14a,b and Figure 15a), EEC models were generated. This EEC model (Figure 15b) is also described in some literature for the oxide and double layer condition [57,58]. The inductive is suggestive of chloride adsorption into metal surface and oxide surface [58,59] or pitting [58,60]. However, this study did not include the inductive loop in the equivalent electrical circuits (EEC) since it was not the main focus, and the value is scattered due to the possibility of the porosity effect in inductive behavior. The Nyquist plot was (Figure 15a) fitted and listed in Table 3. Since the curve showed a depression, the constant phase element (CPE) was used to simulate the non-ideal capacitance behavior, instead of pure capacitance (C). Yo represents the CPE constant while *n* is the model parameter related to deviation, where CPE displays “pure capacitance” behavior if *n* = 1. R_ct_ and Rs show the charge transfer resistance and solution resistance, respectively. The integrity of the oxide layer is given by some parameters, such as, CPE_ox_ as the capacitance of oxide film and R_ox_ as the oxide resistance. However, the physical reasoning of this non-intuitive element is not clear [61,62]. Hence, the CPE was converted to double layer capacitance, *Cdl*, using Equation (1) according to [63].

(1) $$Cdl=Yo{\left(\omega m\right)}^{n-1}$$

where, *ωm* is the frequency at which Zimag reaches its maximum.

In Table 3, a drop in the value of R_ox_ implies that the integrity of the oxide layer was compromised by the Cu-rich phase formation and the rising number of PFZ regions. The APT results in Figure 9a reveal that the Cu-rich phases have a size of at least 20 nm, which is significantly larger than the critical size that can be protected by the aluminum oxide layer (below 8 nm) [64]. Ralston et al. have explained that when the matrix contains Cu intermetallic precipitate exceeding the critical size, it can result in an incomplete passive oxide layer in the vicinity of the particle [64]. This issue is further exacerbated by the high cathodic properties of Cu-containing precipitates, causing the uncovered area surrounding the precipitate to become highly susceptible to local corrosion. Similar challenges are observed in the heat-affected zone (HAZ), which exhibits a lower Cu concentration compared to other regions, leading to a passive layer property closer to pure aluminum. Whereas, Cu in solid solution can help speed up the repassivation process of the layer, which inhibits the pitting growth process. Another study by Y. Kim suggests that local Cu enrichment at incipient pit sites by metastable pitting is possible, which can increase the critical pitting potential under certain circumstance [65]. These mentioned situations are the reasons for the drop in oxide integrity.

All the reasons mentioned above can also be used to explain the differences between the T83 and preheated AM samples. Overall, T83 sample has higher R_ox_ and R_ct_ values compared to the preheated AM samples. The size of PFZ in T83 sample was observed to be smaller than that in the AM samples. According to Wang et al., Al–Cu–Li T8 condition also has PFZ near grain boundaries, but its size is only about 300 nm [20]. This size is narrower than the PFZ observed in the AM at 500 °C, as shown in Figure 5. In addition, the absence of coarse Cu-rich phases in the T83 sample also contributes to higher corrosion resistance. As mentioned earlier, Cu-rich intermetallic phases with low melting point is a characteristic which was predominantly observed in the AM-preheated samples. These results are consistent with previous studies that found negative effects of large PFZ and coarse Cu-rich phase on corrosion resistance [23,57] in Al–Cu–Li alloys. From the results of this study, the key to obtaining a preheated AM 2099 alloy with good corrosion resistance is to control the growth of Cu-rich phase both within and at grain boundaries.

## 4. Conclusions

2XXX series aluminum alloys, such as the AA 2099 Al–Cu–Li, are prone to cracks when printed under the highly non-equilibrium solidification and cooling LPBF conditions. In situ preheating of the build substrate during the print process is beneficial for crack mitigation as well as the triggering of precipitations. In the precipitation of the hardened AA2099 alloy considered in this work, the formation of several competing phases is possible. Of particular interest is the main strengthening plate-like precipitate T_1_ (Al_2_CuLi). The focus of this work was therefore to study how in situ preheating at different temperatures (440 °C, 460 °C, 480 °C, 500 °C and 520 °C) influence the evolution of the precipitation microstructure and its consequential effect on corrosion (open circuit potential, potentiodynamic polarization, Bode and Nyquist plots) and mechanical properties. The results were compared with that of the same alloy but conventionally processed and heat-treated (T83). From our findings, the following conclusions can be drawn.

- As-built microstructure was dominated by coarse columnar grains along the building direction with many intergranular cracks. The as-built microstructure was also characterized by many globular Cu-rich phases both within the grains and on the grain boundaries which tended to be clustered at melt pool boundaries. Calculated dislocation density (3.8949 × 10^13^ m^−2^) was highest in the as-built condition compared to all preheated conditions.
- With preheating at 440 °C, the precipitation microstructure was close to the as-built with the presence of many globular Cu-rich phases dominating the microstructure. However, from 460 °C to 520 °C preheating temperatures, the microstructures were dominated by plate-like and rod-like precipitations suspected to be T_1_ and T_B_, respectively. Increasing preheating temperature was found to influence particle density and size of the precipitates particularly T_1_ in terms of its length.
- As the preheating temperature proceeded further from 500 °C to 520 °C, precipitation-free zones (PFZ) began to widen as more low-melting eutectic Cu-rich phases formed as films around the grain. The remaining globular intragranular Cu-rich phases also coarsen, especially at 520 °C.
- The highest recorded hardness for the AM samples was achieved at 480 °C (93.6 HV0.1), marginally higher than the 460 °C (89.8 HV0.1) and 500 °C (89.9 HV0.1) hardness. The T83 alloy was far superior to the measured hardness of the AM samples. It likewise had superior tensile strength properties compared to the AM (500 °C), although the AM had a better elongation (14.1%).
- The presence of Mg atoms in the characterized T_1_ precipitates in both the AM and the T83 supports the T_1_ nucleation theory that Mg aids in its nucleation. T_1_ plate was found to be shelled by a δ precipitate, a phenomenon which has only previously been reported between δ and Al3TM; TM—transitional metals.
- The potentiodynamic polarization results also demonstrated that increasing preheating temperature increased with the corrosion current density (Icorr) which also indicated higher corrosion rate. The corrosion performance of the conventional T83 alloy was found to be better compared to the AM-preheated alloy.

## Figures and Tables

**Table 1:** Alloy composition of the gas atomized AA2099 Al–Cu–Li powder.

Chemical Composition (wt.%)							
	Al	Cu	Li	Zn	Mg	Mn	Zr
Gas atomized	Bal.	2.63	1.56	0.67	0.28	0.17	0.09
AW 2099-T83	Bal.	2.40	1.60	0.40	0.10	0.10	0.05

**Table 2:** Potentiodynamic polarization parameters of AM-preheated and conventional T83 sample.

Variables	T83	480 °C	500 °C	520 °C
Beta A (V/dec)	0.03 (±0.004)	0.038 (±0.006)	0.055 (±0.007)	0.060 (±0.01)
Ecorr (mV)	−662 (±5)	−643 (±11)	−648 (±8)	−646 (±3)
Icorr (µA/cm^2^)	0.97 (±0.3)	1.31 (±0.6)	5.11 (±1.3)	10.3 (±3)

**Table 3:** Fitting parameters from EIS results based on the EEC in 0.1 M NaCl solution.

Materials	Rs	R_ox_	Yox	*n*	R_ct_	Cdl	*n*
	(KΩ.cm)	(KΩ.cm^2^)	(μS·cm^2^·s)		(KΩ.cm^2^)	(µF.cm)	
T83	0.022	2.91 (±0.5)	32 (±0.06)	0.94	7.71 (±0.7)	0.36	0.89
480 °C	0.016	1.89 (±0.3)	57 (±0.08)	0.95	6.6 (±0.9)	0.6	0.85
500 °C	0.015	1.5 (±0.2)	44 (±0.09)	0.97	4.06 (±0.7)	0.9	0.89
520 °C	0.014	1.25 (±0.1)	34 (±0.09)	0.99	2.78 (±0.02)	0.98	0.9

## Data Availability

Not applicable.

## Supplementary Materials

The following supporting information can be downloaded at: https://www.mdpi.com/article/10.3390/ma16144916/s1, Figure S1: EDS Mapping Showing Cu-enriched phases; Table S1: printing parameters.
