# Supplementary material for: Preheating Influence on the Precipitation Microstructure, Mechanical and Corrosive Properties of Additively Built Al–Cu–Li Alloy Contrasted with Conventional (T83) Alloy
Source: Materials (Basel). 2023 Jul 10;16(14):4916. doi: 10.3390/ma16144916 (PMC10382017; doi:10.3390/ma16144916)
Supplement: Supplementary file 1 [file materials-16-04916-s001.zip › materials-2486934-supplementary.pdf]

materials

MDPI

Supplymentary

Preheating Influence on the Precipitation Microstructure, Mechanical and Corrosive Properties of Additively Built Al–Cu–Li Alloy Contrasted with Conventional (T83) Alloy

Frank Adjei-Kyeremeh [1,*], Yudha Pratesa [2], Xiao Shen [3], Wenwen Song [3,4], Iris Raffeis [1], Uwe Vroomen [1], Daniela Zander [2] and Andreas Bührig-Polaczek [1]

[1] Foundry Institute, RWTH Aachen University, Intzestraße 5, 52072 Aachen, Germany; i.raffeis@gi.rwth-aachen.de (I.R.); u.vroomen@gi.rwth-aachen.de (U.V.); sekretariat@gi.rwth-aachen.de (A.B.-P.)
[2] Chair of Corrosion and Corrosion Protection, Foundry Institute, RWTH Aachen University, Intzestraße 5, 52072 Aachen, Germany; y.pratesa@gi.rwth-aachen.de (Y.P.); d.zander@gi.rwth-aachen.de (D.Z.)
[3] Steel Institute, RWTH Aachen University, Intzestraße 1, 52072 Aachen, Germany; xiao.shen@iehk.rwth-aachen.de (X.S.); song@uni-kassel.de (W.S.)
[4] Institute of Materials Engineering (IfW), University of Kassel, Moencheberg Str. 3, 34125 Kassel, Germany
* Correspondence: f.kyeremeh@gi.rwth-aachen.de

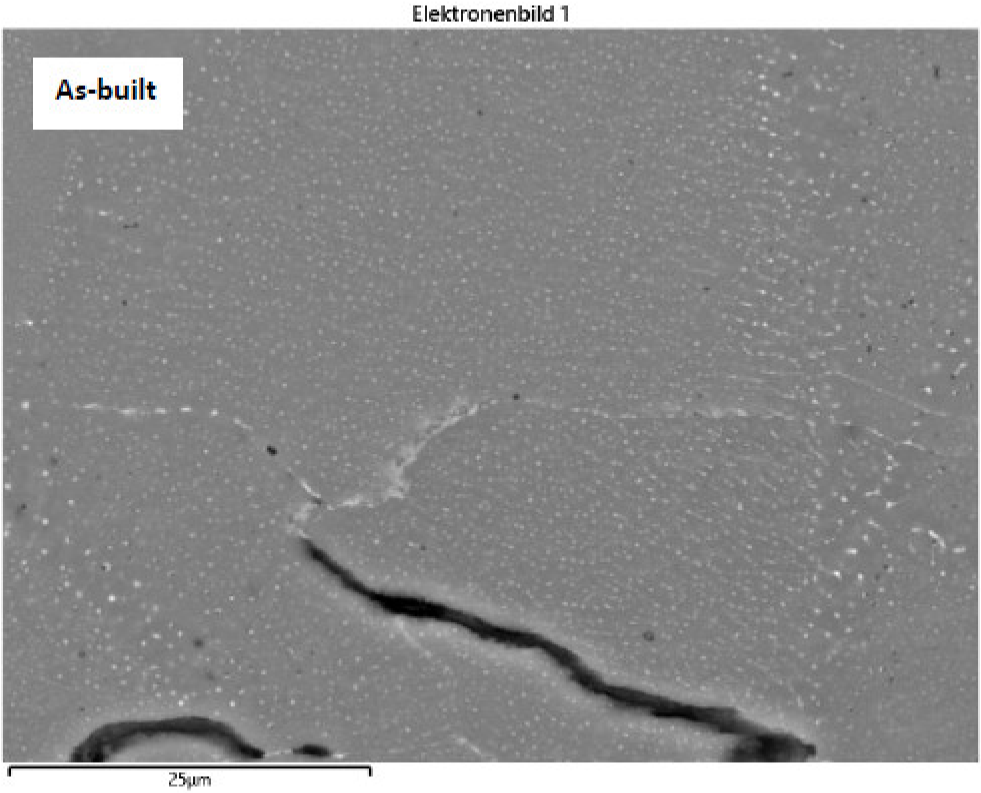
Elektronenbild 1
As-built
25μm

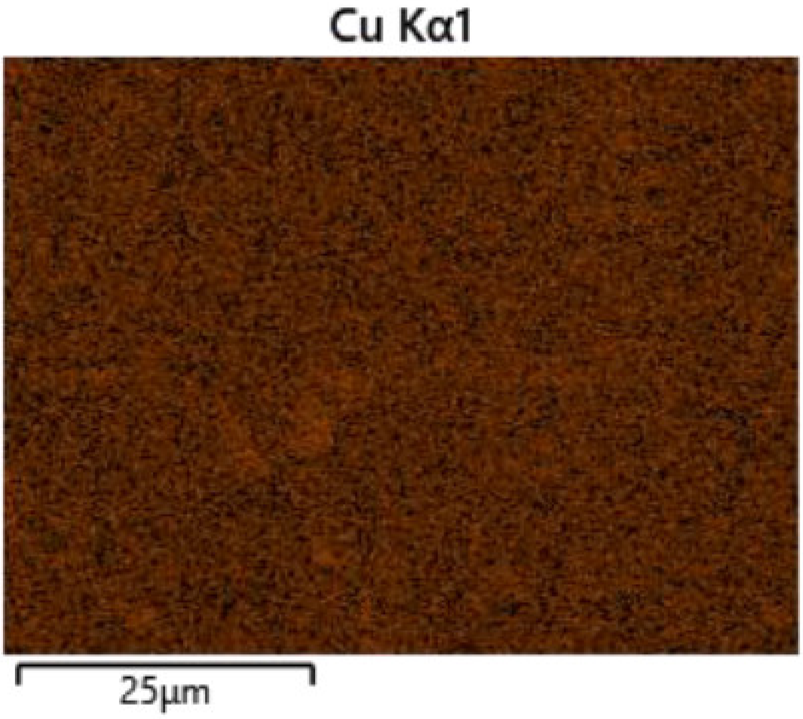
Cu Kα1
25μm

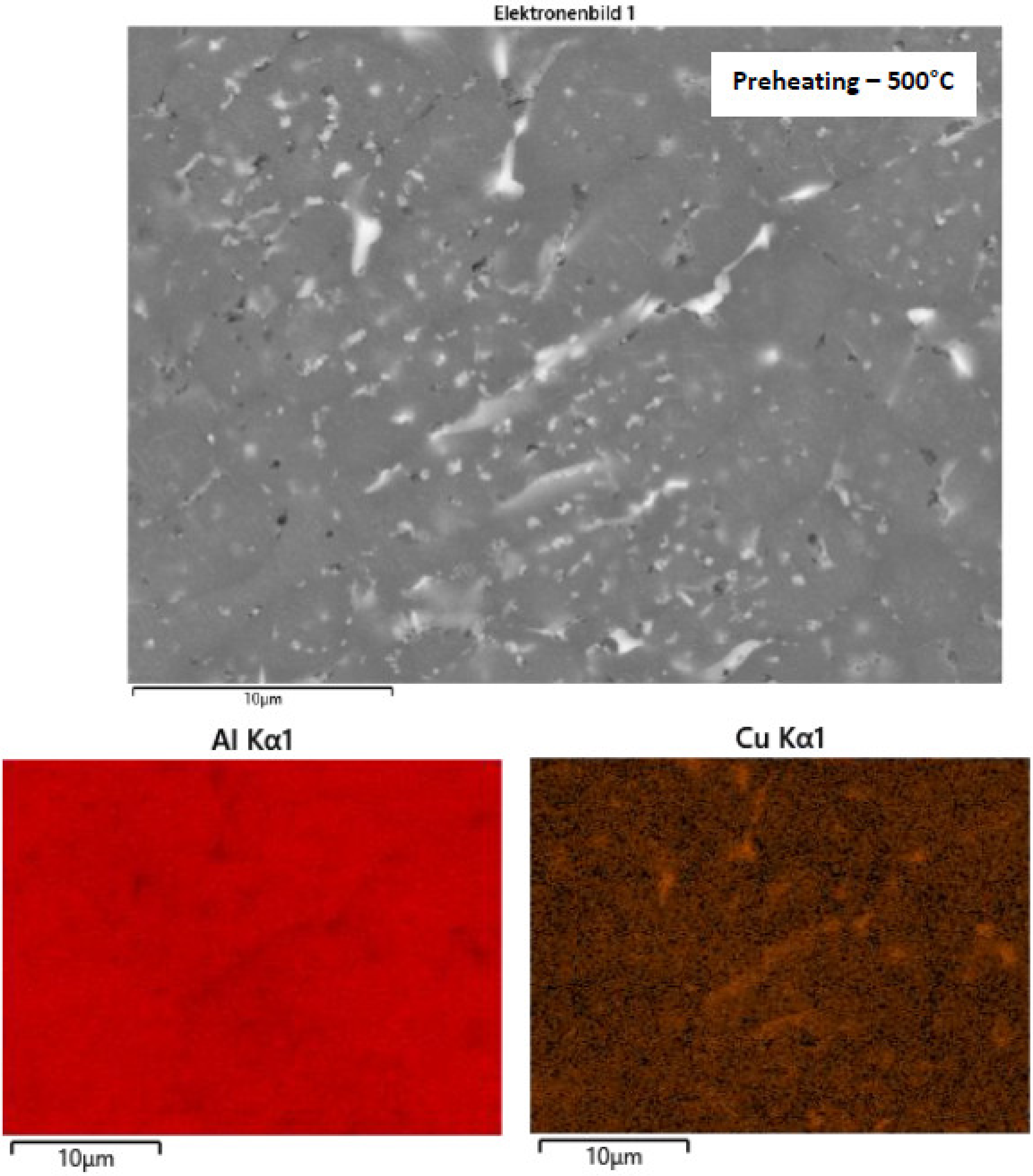

Figure S1. EDS Mapping Showing Cu-enriched phases.

Table S1. printing parameters.

LPBF Process Parameter Design				
	**Laser Power (W)**	**Scan Speed (mm/s)**	**Hatch Distance (μm)**	**Layer Thickness (μm)**
**As-built (RT)**	110	500	140	30
**Preheating °C**				
**440**	90	580	140	30
**460**	90	600	140	30

**480**	100	580	140	30
**500**	90	580	140	30
**520**	90	550	140	30
